# Structure and excitation-dependent emission of novel zinc complexes with pyridyltriazoles†

**DOI:** 10.1039/c9ra02491c

**Published:** 2019-07-16

**Authors:** Alexey Gusev, Elena Braga, Ekaterina Zamnius, Mikhail Kiskin, Mariya Kryukova, Alina Baryshnikova, Boris Minaev, Gleb Baryshnikov, Hans Ågren, Wolfgang Linert

**Affiliations:** N.S. Kurnakov Institute of General and Inorganic Chemistry, Russian Academy of Sciences Moscow 119991 Russia galex0330@gmail.com; Institute of Chemistry, Saint Petersburg State University Universitetskaya Nab. 7/9 Saint Petersburg 199034 Russia; Department of Chemistry and Nanomaterials Science, Bohdan Khmelnytsky National University 18031 Cherkasy Ukraine; Division of Theoretical Chemistry and Biology, School of Engineering Sciences in Chemistry, Biotechnology and Health, KTH Royal Institute of Technology 10691 Stockholm Sweden; Tomsk State University 36 Lenin Avenue Tomsk Russia; Institute for Applied Synthetic Chemistry, Vienna University of Technology Getreidemarkt 9/163 A-1060 Vienna Austria

## Abstract

A series of Zn(ii) complexes with 5-(4-*R*-phenyl)-3-(pyridin-2-yl)-1,2,4-triazoles have been synthesized and subsequently characterized by single crystal X-ray diffraction, ^1^H-NMR, FT-IR spectroscopy, elemental analyses, ESI-MS, and PXRD. The X-ray diffraction analyses revealed that the complexes have a similar molecular structure and their supramolecular frameworks are constructed by hydrogen bonds and π⋯π interaction scaffolds. Upon irradiation with UV light, the studied complexes display deep blue emission at 396–436 nm in the solid state. The compounds show an unexpected excitation-dependent emission phenomenon which is detected by a change in the emission color (from blue to yellow) upon increase of the excitation wavelength. The conducted quantum-chemical calculations indicate that supramolecular differences in the single-crystal architecture of the synthesized complexes play a crucial role for this photophysical behaviour.

## Abstract 2

A series of Zn(ii) complexes with 5-(4-*R*-phenyl)-3-(pyridin-2-yl)-1,2,4-triazoles have been synthesized and subsequently characterized by single crystal X-ray diffraction, ^1^H-NMR, FT-IR spectroscopy, elemental analyses, ESI-MS, and PXRD.

## Graphical abstract

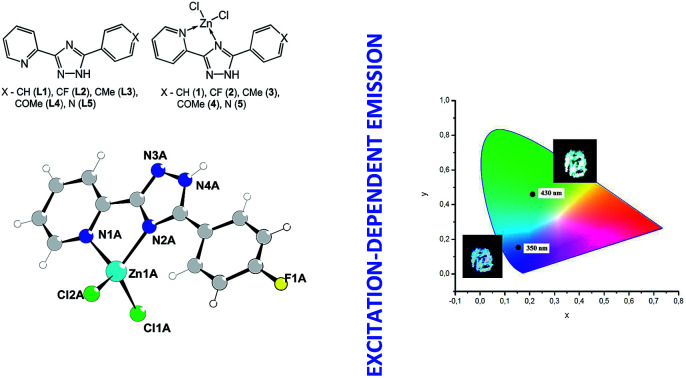

## Introduction

Organo–metallic compounds attract increasing attention in the emerging field of organic electronics. This is due to their potential applications in optical and optoelectronic devices such as light-emitting diodes, optical wave guides and optically pumped lasers.^1^ For the applications of luminescent complexes in optoelectronic devices it is highly desirable to be able to tune emission intensity and color.^2^ However, variation of the solid state luminescence color is less achievable than in solution due to the difficulty of obtaining different stable phases in the aggregated state with varied molecular conformations.^3^ The design and synthesis of organic–inorganic hybrid complexes based on strong coordination bonds and multiple weak non-covalent interactions have in recent times become a rapidly developed research field^4^ since the incorporation of well-ordered structural components into crystal lattices may lead to new advanced materials with desired properties. It has been found that all the tunable chromaticity variations are usually associated with non-covalent interactions in global (supramolecular) crystal packing, like hydrogen bonds, π⋯π, C–H⋯π contacts and/or other intermolecular linkers.^5^ Noticeable changes in the photophysical characteristics are also frequently observed for different polymorphs of the same compound.^5^ More conspicuous is the fact that the luminescent complexes display sharp variations in the emissive properties while maintaining the same crystal structure. Only a few examples of such chromic behavior have been reported so far and a definitive explanation is still lacking.^3,6^

Pyridylazole ligands are frequently used in coordination chemistry due to their significant ability to form stable complexes with metal ions.^7^ Zinc azolate luminescent complexes can be used as active optoelectronic materials and as probes in biological systems.^8^ Controllable tuning of fluorescent properties of the zinc complexes based on ligand design always represents a challenge for chemists. From the point of view of molecular and crystal engineering, the critical criterion for ligand design is how to embed non-covalent interacting groups to control the molecular structure and the supramolecular framework. Surprisingly, despite the fact that Zn(ii) complexes with 1,2,4-triazole ligands demonstrate excellent luminescent properties in the solid phase their crystal structure peculiarities and photophysics have been only fragmentary studied.^8*e*^ One of the reasons for this situation is the synthetic difficulties associated with obtaining crystalline samples requiring the use of solvothermal methods.

**Scheme 1:**
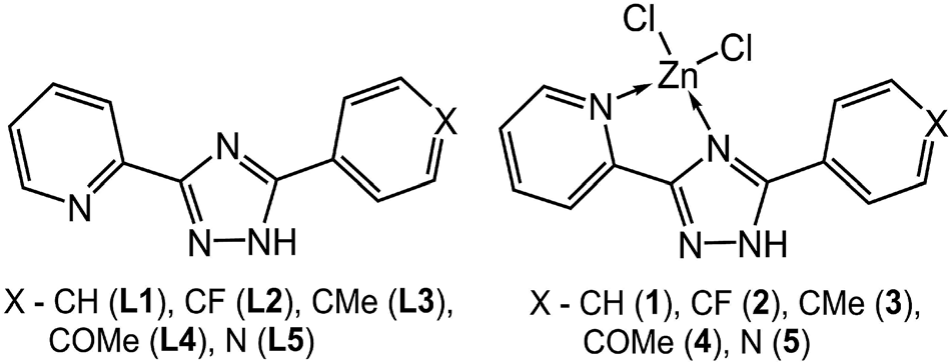


In line with the above discussion and the fact that the type of substituents is a main factor affecting the supramolecular and luminescent properties, five pyridyltriazole ligands (Scheme 1), carrying different substitutions have been employed for the synthesis of novel Zn(ii) complexes. In this paper, we report an interesting emissive behavior of five studied complexes with pyridyltriazole ligands in the solid state (Scheme 1). The electronic effects of the substituents on the excitation-dependent properties were also determined as important factor for the modulation of the luminescence for the studied systems. We have carried out quantum-chemical calculations for a better understanding and explanation of the unusual phenomenon of excitation wavelength-dependent solid state fluorescence of the studied complexes (2–4) in contrast to the other two synthesized compounds (1 and 5).

## Experimental section

### Materials and methods

All the reagents and solvents were commercially available and used as received without further purification. The L1–L5 ligands were synthesized as described in the literature.^9^ For more detail see ESI.† Elemental analyses of C, H, and N were performed with the EuroEA 3000 analyzer.

The IR spectra were measured by the FSM 2202 spectrometer in the range of 4000–400 cm^−1^. ^1^H NMR spectra were recorded on a Bruker VXR-400 spectrometer at 400 MHz using the DMSO-d^6^ solutions. Absorption spectra were recorded with a Cintra-3000 spectrometer. Photoluminescence spectra of solid state samples were recorded on the FluoroMax-4 spectrometer. The quantum yields of the solid samples were determined by the absolute method using an integrating sphere. Fluorescence decay lifetimes were measured using a Picoquant 300 TCSPC.

### Procedure for the preparation of the zinc complex

All complexes were obtained by the similar methods so only synthesis of complex 1 is described in details.

To suspension of 222 mg (1 mmol) 5-(pyridine-2-yl)-3-phenyl-1,2,4-triazole (L1) in 25 mL of anhydrous acetonitrile a mmol amount of ZnCl_2_ (133 mg) was added under stirring. The mixture was stirred and heated under reflux conditions for 1 h during which a white solid precipitate was formed. Then the mixture was cooled to the room temperature and filtered. Bulk crystals were recrystallized from CH_3_CN solvent and colorless crystals of complex 1 were obtained after 3 days.

#### Complex 1

Yield: 245 mg (69%). Anal. calcd (%) for [C_13_H_10_Cl_2_N_4_Zn] (M = 358.53 g mol^−1^): C, 43.55; H, 2.81; N, 15.62. Found: C, 43.72; H, 2.61; N, 15.58. FT-IR bands (cm^−1^): 3094(w), 1615(s), 1498(s), 1474(s), 1387(s), 793(m), 722(s), 683(s), 412(w). ^1^H NMR (400 MHz, DMSO-d^6^): *δ* 14.84 (s, 1H), 8.73 (s, 1H), 8.19 (d, 1H), 8.11 (m, 2H), 8.04 (dt, 1H), 7.52 (m, 4H). ESI-MS (positive) in methanol: the base mass-spectral peak was detected at *m*/*z* = 320.98 corresponding to the [ZnL_1_Cl]^+^ species.

#### Complex 2

Yield: 214 mg (57%). Anal. calcd (%) for [C_13_H_9_Cl_2_FN_4_Zn] (M = 376.52 g mol^−1^): C, 41.47; H, 2.41; N, 14.88. Found: C, 41.52; H, 2.32; N, 14.71. FT-IR bands (cm^−1^): 3073(w), 1611(s), 1501(s), 1470(s), 1386(s), 843(m), 760(s), 741(s), 413(w). ^1^H NMR (400 MHz, DMSO-d^6^): *δ* 14.82 (s, 1H), 8.73 (s, 1H), 8.20 (d, 1H), 8.10 (d, 2H), 8.04 (dd, 1H), 7.52 (m, 3H). ESI-MS (positive) in methanol: the base peak was detected at *m*/*z* = 338.97 corresponding to the [ZnL_2_Cl]^+^ species.

#### Complex 3

Yield: 278 mg (75%). Anal. calcd (%) for [C_14_H_12_Cl_2_N_4_Zn] (M = 372.55 g mol^−1^): C, 45.13; H, 3.25; N, 15.03. Found: C, 45.32; H, 3.39; N, 14.94. FT-IR bands (cm^−1^): 3067(w), 1612(s), 1501(s), 1464(s), 1381(s), 821(m), 756(s), 736(s), 416(w). ^1^H NMR (400 MHz, DMSO-d^6^): *δ* 14.72 (s, 1H), 8.71 (s, 1H), 8.17 (d, 1H), 8.04 (dd, 1H), 7.99 (d, 2H), 7.56 (dd, 1H), 7.33 (d, 2H), 2.37 (s, 3H). ESI-MS (positive) in methanol: the base peak was detected at *m*/*z* = 335.00 corresponding to the [ZnL_3_Cl]^+^ species.

#### Complex 4

Yield: 295 mg (76%). Anal. calcd (%) for [C_14_H_12_Cl_2_N_4_OZn] (M = 388.55 g mol^−1^): C, 43.27; H, 3.11; N, 14.42. Found: C, 43.51; H, 3.16; N, 14.28. FT-IR bands (cm^−1^): 3076(w), 1606(s), 1499(s), 1470(s), 1388(s), 840(m), 751(s), 716(s), 415(w). ^1^H NMR (400 MHz, DMSO-d^6^): *δ* 14.68 (s, 1H), 8.71 (s, 1H), 8.17 (d, 1H), 8.04 (m, 3H), 7.56 (dd, 1H), 7.08 (d, 2H), 3.82 (s, 3H). ESI-MS (positive) in methanol: the base peak was detected at *m*/*z* = 350.99 corresponding to the [ZnL_4_Cl]^+^ species.

#### Complex 5

Yield: 211 mg (59%). Anal. calcd (%) for [C_12_H_9_Cl_2_N_5_Zn] (M = 359.52 g mol^−1^): C, 40.08; H, 2.52; N, 19.48. Found: C, 40.14; H, 2.94; N, 19.44. FT-IR bands (cm^−1^): 3082(w), 1613(s), 1509(s), 1462(s), 1386 (s) 1023(m), 846(m), 751(s), 723(s), 413(w). ^1^H NMR (400 MHz, DMSO-d^6^): *δ* 14.94 (s, 1H), 8.72 (m, 3H), 8.18 (d, 1H), 8.04 (m, 3H), 7.59 (t, 1H). ESI-MS (positive) in methanol: the base peak was detected at *m*/*z* = 322.01 corresponding to the [ZnL_5_Cl]^+^ species.

### Crystallography

The single crystal X-ray diffraction data for 1 and 3 were collected using the Bruker APEX II diffractometer equipped with a CCD detector and a graphite-monochromated Mo Kα radiation source (*λ* = 0.71073 Å). The single crystal X-ray diffraction data for 2 and 5 were collected using the SuperNova diffractometer equipped with a HyPix-3000 detector and a micro-focus Cu Kα radiation source (*λ* = 1.54184 Å). The structures of complexes were solved by the direct methods and refined in the full-matrix anisotropic approximation for all non-hydrogen atoms. The hydrogen atoms of water molecule were found in differential Fourier maps and their parameters were refined using the riding model. The hydrogen atoms of the carbon-containing ligand were positioned geometrically and refined by using a riding model. All the calculations were performed by direct methods and using the SHELX-2014 program package.^10^ The crystallographic parameters and the structure refinement statistics for 1–3 and 5 are shown in Table S1.†

### Computational details

The single point (SP) calculations of [1]_9_, [2]_11_ and [3]_10_ crystal fragments extracted from the single-crystal X-ray diffraction data were performed at the B3LYP/6-31G(d) level of theory.^11^ After that the topological analysis of electron density distribution by the Bader “Atoms in Molecules” (QTAIM)^12^ method was performed in order to identify the electronic peculiarities of all non-covalent interactions responsible for the binding of each molecule in the single crystal. The shapes of the frontier molecular orbitals were computed at the same B3LYP/6-31G(d) level of theory within a control value of isosurface equal to 0.03 a.u. The energy, intensity and electronic configuration of the first excited singlet state (S_1_) for the studied complexes 1–3 were calculated at the time-dependent (TD) density functional theory (DFT)^13^ level using the same B3LYP/6-31G(d) approach and gas phase approximation. Accounting for the fact that the S_1_ state was found to be of charge-transfer (CT) nature the long-range corrected CAM-B3LYP functional^14^ was also used for the estimation of the S_1_ state energy. All the DFT calculations were performed using the Gaussian 16 program package.^15^ The QTAIM calculations were carried out using AIMAll package.^16^

## Results and discussion

### Synthesis and spectral characterization

**Scheme 2:**
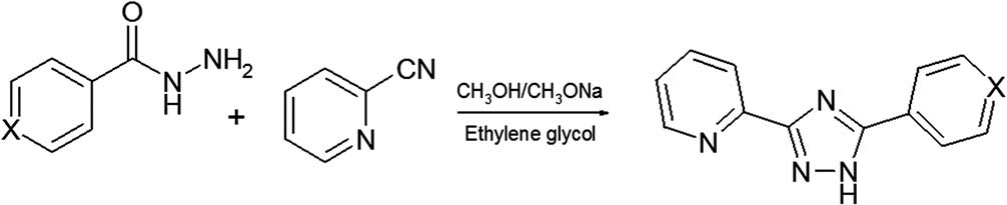


The ligands L1–L5 were prepared by a two-step reaction of 2-cyanopyridine and the appropriate hydrazide as shown in Scheme 2. The complexes ZnLCl_2_ were obtained by reaction of the corresponding ligands with ZnCl_2_ in acetonitrile. The zinc complexes were characterized by elemental analysis, IR-, ^1^H-NMR spectroscopy and mass spectrometry (Fig. S1–S3†). Additionally, to confirm the phase purity and stability of complexes, the original samples were characterized by PXRD.

The FTIR spectra of ligands and complexes confirm the molecular form of the ligands and coordination *via* pyridine and triazole nitrogen atoms. The infrared spectra of these five Zn(ii) complexes are similar to that of the corresponding ligand and the IR assignments of the selected characteristic bands. In the IR spectra of complexes 1–5, the existence of the C=N bonds from pyridine and triazole rings provides the strong characteristic peaks in the short ranges at 1606–1615 and 1498–1509 cm^−1^, respectively. These bands undergo negative shifts in the complexes comparable with the free ligands, which is caused by the coordination of the nitrogen atoms to the metal ions. This is further confirmed by appearance of the new *ν*(M–N) vibrations in the region 413–416 cm^−1^ in all studied complexes.

The ^1^H NMR spectra of the complexes also confirm the coordination structure of the studied complex species. The remarkable NH-proton resonances are found at 14.68–14.94 ppm which are shifted downfield comparing with the free ligands indicating that triazole core is stills protonated after the complexation with zinc. The total set of aromatic proton signals and the nature of corresponding splitting ratios generally coincide with those for the free ligands except slight downfield shift due to coordination between the nitrogen atoms and the Zn(ii) center.

The ESI-mass spectrometry was used to further verify the composition of the complexes in acetonitrile solution. The results suggest that two main forms of the complexes exist in solution. The most intense peaks correspond to the molecular weight of ZnLCl_2_ and ZnL_2_Cl_2_ species. It is noteworthy that only complexes with a 1 : 1 ratio of zinc and ligand do crystallize in the solid state.

To further understand the structures of these complexes, single crystals were obtained and analyzed by single crystal X-ray diffraction. Unfortunately, recrystallization in all cases led to the formation of low quality crystals due to the formation of twins. Thus, the complete single-crystal X-ray analysis was done for the complexes 1, 2, 3 and 5. The phase purity of the bulk samples was confirmed by XRD analysis.

### Description of the structures

**Fig. 1:**
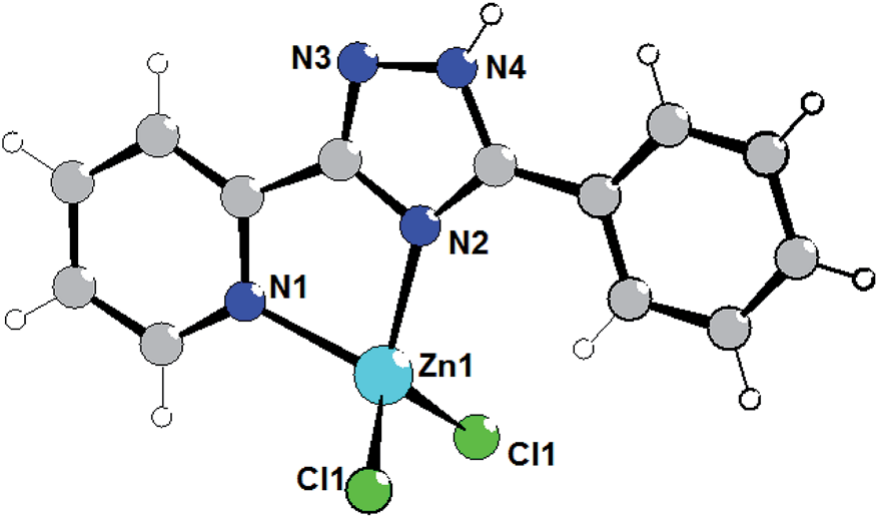
Structure of complex 1. Selected bond lengths (Å) are: Zn1–N2 2.059(6), Zn1–N1 2.066(6), Zn1–Cl1 2.2049(14).

The isostructural complexes 1 and 5 are crystallized in the monoclinic space group *P*2_1_/*m*. As shown in Fig. 1, the asymmetric unit of complex 1 consists of one Zn(ii) atom, one L1 ligand and two chlorine anions. The Zn(ii) cation is bound to one triazolyl N atom (N2) and one pyridyl N atom (N1) of triazole ligand (Fig. 1).

The average lengths of Zn–N and Zn–Cl bonds are 2.063(6) and 2.205(2) Å, respectively. The bond angles around the Zn(ii) ion are in the range of 81.2(2)–115.30(8)°. It is notable that the Zn–N_triazole_ bonds are almost equivalent to the Zn–N_pyridine_ bonds, which is non-typical for coordination chemistry of (pyridin-2-yl)-1,2,4-triazoles.^7^ Using Addison's model^17^ the coordination geometry around the zinc atom in complex 1 (*τ* = 0.918) can be described as an almost perfect tetrahedron. One can note that the pyridine, phenyl and triazole rings are perfectly coplanar.

**Fig. 2:**
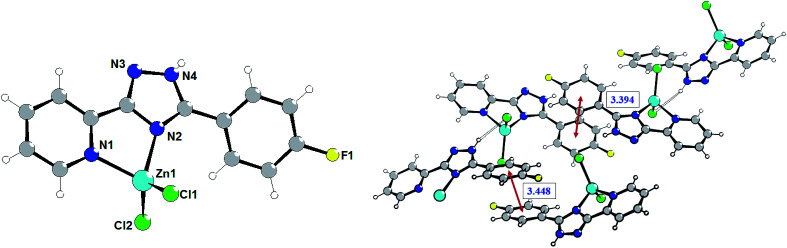
Molecular structure of complex 2 (left) and a fragment of the crystalline lattice of 2 (right). Selected bond lengths (Å) are: Zn1–Cl1 2.218(3), Zn1–Cl2 2.195(3), Zn1–N2 2.035(7), Zn1–N1 2.079(7). The π⋯π stacking interactions are shown by red arrows.

The crystal refinement data of complex 2 implies that it belongs to a triclinic system, *P*1̄space group. The asymmetric unit consists of two crystallographically and conformationally independent molecules. The zinc atom is in a four-coordinate environment composed of the two halid ligands and two nitrogen atoms of the ligand L2 (Fig. 2). As expected, the coordination geometry of the zinc cation represents a distorted tetrahedron, with a four-coordinated geometry index *τ*_4_ = 0.905 (Zn1) and 0.898 (Zn1A). Bond lengths and angles for each zinc atom are comparable to those found in previously described complexes with an analogous environment.^8*e*^ The dihedral angles between the triazolyl ring and the phenyl ring in the two molecules are 18.83° and 30.48°, respectively. Single crystal analysis shows that the main difference between structures 1 and 2 is in the feature of crystalline packing. The crystal structure of complex 2 reveals that the independent complex 2 species are linked through the N4–H4⋯Cl1A and N4A–H4A⋯Cl1 hydrogen bonds interactions (the distance of H4A⋯Cl1 is 2.352 Å, and the N4A–H4A⋯Cl1 angle is 150.05°) to generate a one-dimensional “wave-like” chain. In contrast to complex 1, complex 2 possesses additional intermolecular π⋯π stacking interactions that affect significantly its supramolecular structure; π_phenyl_⋯π_phenyl_ stacking interactions between fluorophenyl rings separated of about 3.394 and 3.448 Å, respectively, leads to the binding of two H-bonded chains in the double-chain.

**Fig. 3:**
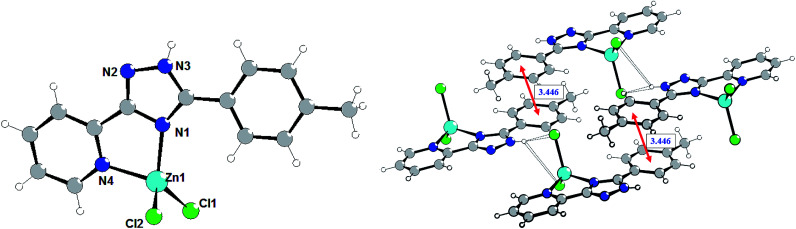
Molecular structure of complex 3 (left) and a fragment of the crystalline lattice of 3 (right). Selected bond lengths (Å) are: Zn1–N1 2.0406(14), Zn1–N4 2.0695(14), Zn1–Cl1 2.1920(6), Zn1–Cl2 2.2398(6). The π⋯π stacking interactions are shown by red arrows.

The crystal refinement data of complex 3 implies that it belongs to a triclinic system, *P*1̄ space group. As shown in Fig. 3, the Zn(ii) center adopts a distorted tetrahedral geometry (*τ*_4_ = 0.929) coordinated by two nitrogen atoms of 5-(pyridine-2-yl)-3-(4-methylphenyl)-1,2,4-triazole (L3) and two terminal chloride ions. The bond angles for Zn(ii) are in the range of 81.65(6)–116.64(4)°. The dihedral angle between the pyridyl-triazole moiety and the phenyl ring is 13.8°. The separate species of complex 3 are linked through the N3–H3A⋯Cl2 hydrogen bonds interactions (the distance of H3A⋯Cl2 is 2.424 Å, and the N3–H3A⋯Cl2 angle is 160.78°) to generate a one-dimensional chain, that are further aggregated in a double-chain (Fig. 3) by the π⋯π stacking interactions with the centroid⋯centroid distances of 3.446 Å. Two adjacent double-chains are interconnected *via* the C14–H9⋯Cl1 hydrogen bond interactions (H9⋯Cl1 = 2.832 Å) forming a 2D layer.

### Non-covalent interactions and QTAIM analysis

As follows from the QTAIM analysis the crystal packing of all studied complexes is characterized by the highly-developed network of non-covalent interactions. All of them can be classified by the following groups: (1) π-stacking C⋯C, C⋯N, CH⋯C and CH⋯N interactions; (2) side hydrogen bonds involving Cl atoms and NH groups. (3) long-distance interactions of different types including the H⋯H contacts. All these bonds correspond to the closed-shell type interactions (*h*_e_(*r*) > 0, ▽2*ρ*(*r*) > 0 in the corresponding critical points of the (3, −1) type) (Tables S2–S4†). The calculated Laplacian values and electron density *ρ*(*r*) values for the first and second group of bonds coincide with the Koch–Popelier average range (0.024–0.139*ea*_0_^5^ and 0.002–0.035*ea*_0_^3^, respectively) which is commonly used as a criterion of the real existence of non-covalent interactions. The remaining part of the non-covalent bonds (third group) that do not satisfy the Koch–Popelier criteria are characterized by very small values of the Laplacian, electron density and potential energy density *ν*(*r*) in the corresponding (3, −1) critical points. Together with the high ellipticity it indicates an existence of these bonds only upon fixed positions of the atomic nuclei. In fact, these bonds are only statically stable, but in a real crystal they are dynamically unstable and formally do not exist. Surprisingly, that the total concentration of potential energy density *ν*(*r*) in the intermolecular space per one molecule of the complex (Tables S2–S4†) is almost the same for the crystal packing fragments [1]_9_, [2]_11_ and [3]_10_ (−0.103, −0.104 and −0.107 a.u., respectively). It means that the energy of these non-covalent infarctions (*E*_int_) is also almost the same applying the simple Espinosa equation (*E*_int_ = 0.5 *ν*(*r*)).^18^ Thus, we can conclude that the excitation-dependent behaviour of the emission maximum for the compounds 2–4, in contrast to compounds 1 and 5, is not caused by the different strength of non-covalent interactions for their single crystals. Most probably the origin of this interesting phenomenon lies in the different architecture of the single crystals. Really, the crystals of compound 1 and 5 are of the layered architecture and they contain just one type of π-stacking dimers due to the interaction of pyridyltriazole fragments (type I). While the crystals of compounds 2–4 contain at the same time two types of π-stacking dimers: the less-coupled type I dimers and the strongly-coupled type II dimers, due to the interaction of phenyl rings. Thus, we can predict that just only type II dimers are responsible for the excitation-dependent emission behaviour of 2–4 crystals discussed in the next sections.

### Photophysical studies

**Fig. 4:**
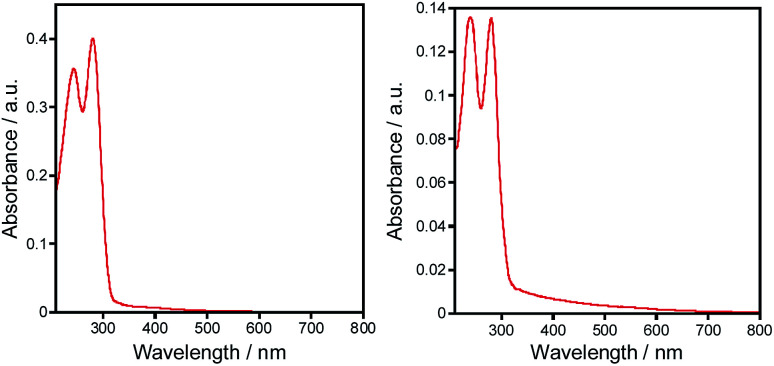
UV-vis spectra of complexes 1 (left) and 2 (right) in solid state.

For the obtained complexes 1–5 we have been particularly interested in the photophysical properties. The UV/vis spectra of complexes 1–5 were investigated on the ground of diffuse reflectance spectra of the solid samples. It was found that all the complexes exhibit two distinct absorption bands at *ca.* 273–284 nm and 240–246 nm that correspond to the π–π* and n–π* transitions within aryl and triazole groups, respectively (Fig. 4). These bands are red shifted compared to those of the free ligands.

**Fig. 5:**
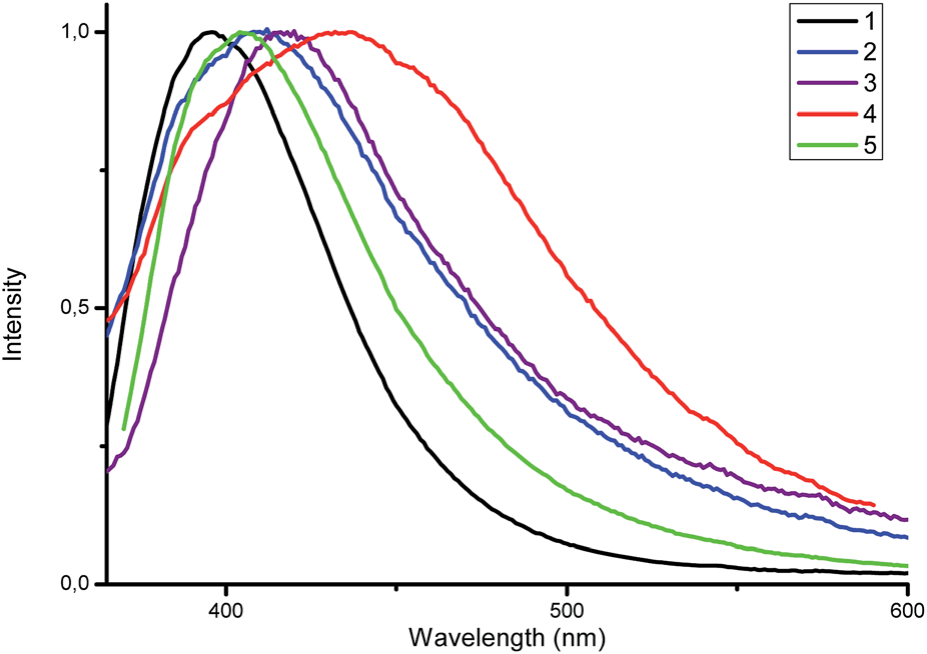
Normalized emission spectra of 1–5 in the solid state.

Luminescent zinc(ii) complexes possessing closed 3d^10^ electronic shells are superior potential candidates as valuable luminescent materials; thus, we probed the luminescence properties of 1–5 in the solid state at room temperature. Selected data are summarized in Table 1. As shown in Fig. 5, upon the excitation at 350 nm complex 1 displays strong emission with a maximum at 396 nm. For the complexes 2–5 the emission bands are centered at 408, 418, 436 and 404 nm, respectively, which are consistent with the electron-donating ability of the substituents (–F < –CH_3_ < –OCH_3_). The electron-donating effect decreases the energy difference between HOMO and LUMO, leading to the red shift of the emission maximum (*λ*_em_).^19^

**Table 1:** Photophysical properties of complexes 1–5

Complex	*λ*_em_ (nm)	*τ* (ns)	QY (%)	CIE
1	396	1.50	39.8	0.15; 0.03
2	408	1.23; 4.92	28.3	0.17; 0.12
3	418	2.18; 8.73	7.5	0.16; 0.11
4	436	2.04; 8.19	11.3	0.16; 0.12
5	404	1.74	30.6	0.16; 0.07

All studied complexes exhibit deep blue luminescence emission - the CIE coordinates are summarized in Table 1. In addition, the emission bands of 1–5 are red-shifted compared to those of the corresponding ligands; this is caused by the effect of coordination.

The luminescence quantum yield (QY) of complexes 1–5 in the solid state were measured by the absolute method using an integrating sphere (Table 1). The highest quantum yield was obtained for complex 1 (39.8%). Introduction of substituent in *para*-position leads to a reduction of luminescent efficiency. It is noteworthy that the luminescence intensities for the complexes 1 and 5 are stronger than those for the other complexes, which may be related to the better planarity and the absence of intermolecular interactions for the assembled supramolecular structures in the solid state complexes.

Generally, the quantum yields of complexes 1–5 (6–40%) are much higher than those of the corresponding ligands (QY < 1%); which can be explained by the increased rigidity of the complexed ligand affecting suppression of non-radiative quenching processes within the complex species.^5^ It is also worthwhile to note that the solid-state quantum yields of 1 and 5 are high enough to apply these materials as emitting materials in OLEDs.^1^

The luminescence decay profiles of the 1–5 complexes were measured at 350 nm excitation wavelengths in the solid state at 298 K. The detailed data are listed in Table 2. Complexes 2–4 display bi-exponential decays, while the complexes 1 and 5 show mono-exponential decay fits. The calculated lifetime lies in the nanosecond range, which is typical for fluorescent emission. The multi-exponential decay lifetimes indicate population of long-lived excited states (like excimers) along with the main (prompt) emissive channel (Table 2).

**Table 2:** Photoluminescent parameters of complexes 2–4 at different excitation wavelengths

Complex 2				Complex 3				Complex 4			
*λ*_ex_ (nm)	*λ*_em_ (nm)	CIE	QY (%)	*λ*_ex_ (nm)	*λ*_em_ (nm)	CIE	QY (%)	*λ*_ex_ (nm)	*λ*_em_ (nm)	CIE	QY (%)
430	513	0.27; 0.47	8.03	450	563	0.41; 0.54	8.10	430	515	0.30; 0.52	2.46
420	491	0.24; 0.38	10.72	420	527	0.32; 0.47	9.32	420	507	0.31; 0.48	3.38
410	492	0.23; 0.34	13.50	410	522	0.29; 0.42	8.89	410	491	0.25; 0.39	4.44
400	482	0.21; 0.32	13.81	400	493	0.27; 0.39	7.91	400	477	0.21; 0.33	6.64
390	471	0.21; 0.28	13.89	390	483	0.25; 0.35	6.35	390	474	0.19; 0.24	6.51
380	465	0.2; 0.23	17.58	380	468	0.21; 0.28	5.35	380	468	0.18; 0.198	6.33
370	438	0.18; 0.18	23.84	370	431	0.19; 0.197	5.15	370	465	0.17; 0.18	6.96
360	423	0.18; 0.145	26.02	360	421	0.17; 0.14	5.98	360	453	0.16; 0.14	7.99
350	408	0.17; 0.12	28.32	350	420	0.16; 0.11	7.45	350	432	0.16; 0.12	11.31

### Excitation-dependent emission

The most interesting results were obtained by studying the photoluminescence of solid samples upon different excitation wavelengths. As mentioned above, the blue luminescence of complexes 1–5 was excited by radiation with a wavelength of 350 nm. To our surprise, we accidentally discovered a weak greenish luminescence when the complex 4 was irradiated by a blue lamp. Similar emission was detected for complexes 3 and 4, while complexes 1 and 5 did not emit in these conditions. This fact encouraged us to investigate the fluorescence of the complexes 2–4 in greater detail *via* excitation with other wavelengths.

**Fig. 6:**
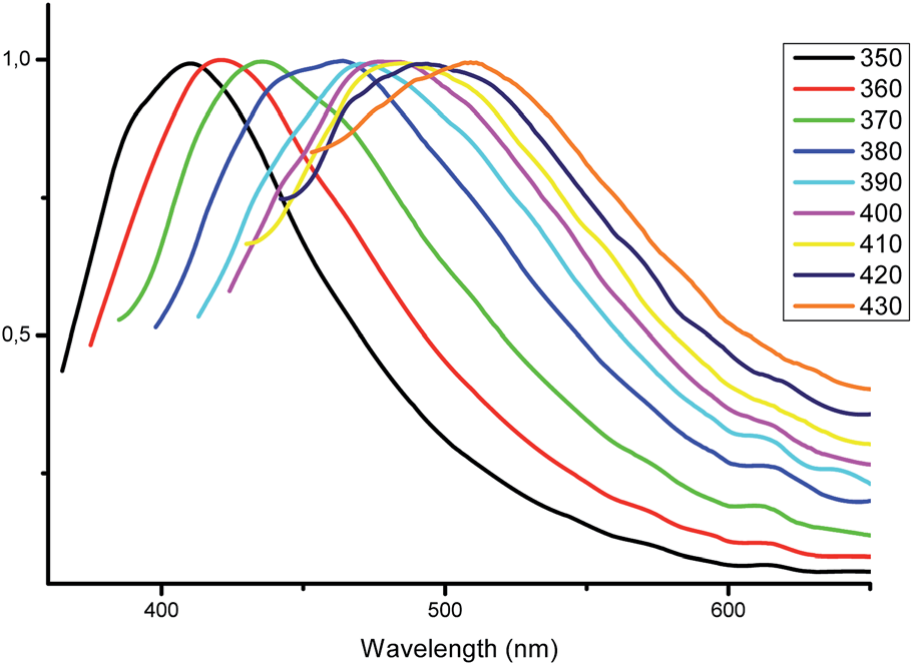
Normalized emission spectra of complex 2 in the solid state at different excitation wavelengths.

With excitation wavelengths below 350 nm, only a minor shift in the maximum emission wavelength of 408 nm is observed for complex 2. When the wavelength of excitation light increases from 370 to 430 nm, the emission maximum also gradually shifts from 423 to 513 nm (Fig. 6). Even upon excitation at 440 nm the quantum yield of complex 2 is 8%, which is a pretty satisfactory result for fluorescent zinc complexes in the solid state and makes such complexes promising optical materials.

**Fig. 7:**
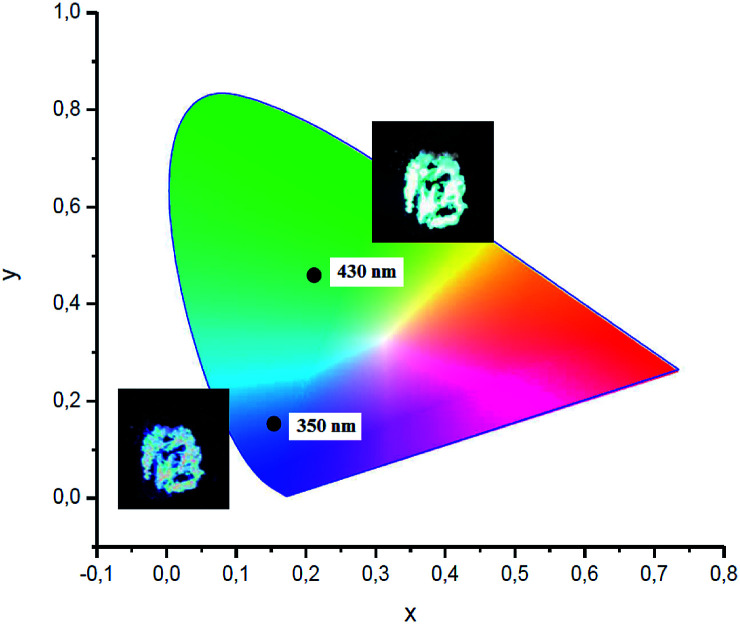
CIE 1931 chromaticity diagram and photo of complex 2 excited at 350 and 430 nm.

Visually, the excitation shift is accompanied by a shift of the color of the emitting complex 2 from blue to greenish yellow (Fig. 7). The corresponding CIE coordinates are also presented in Fig. 7. As the excitation wavelength exceeds 440 nm, the emission intensity decreases rapidly without noticeable maximum shift. Compounds 3 and 4 also possess the excitation wavelength-dependent emissive property. With the increase of the excitation wavelength from 350 to 450 nm the emission maximum gradually shifts from 418 to 561 nm for complex 3 (Fig. S1†) and from 432 to 515 nm for complex 4 (Fig. S2†).

It should be noted that the observed phenomenon is extremely rare in the literature.^20^ Although understanding mechanisms of photochemical reactions as well as transform a low contrast fluorochromic material into a high contrast one by selecting different wavelengths of excitation light, as well as enabling the multichannel responses present important challenges in chemistry.

### Theoretical interpretation of excitation-dependent emission

**Fig. 8:**
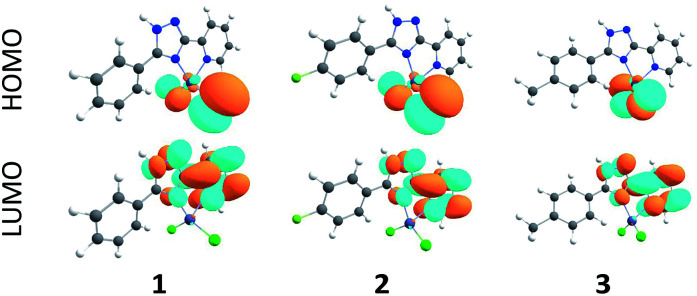
Frontier molecular orbitals for the complexes 1–3 calculated by the B3LYP/6-31G(d) method.

As follows from our TDDFT calculations, all the studied single–molecule complexes are characterized by the same HOMO–LUMO electronic configuration of the S_1_ state. As can be seen from Fig. 8, the HOMO orbital is strongly localized at the Cl ligands (n-orbitals) with only a small admixture of d-function on the Zn central atom. At the same time, LUMO wave functions represent typical π-orbitals localized on the triazole–pyridine fragment. This is the reason that the HOMO–LUMO excitation can be classified as a charge-transfer (CT) electronic transition. The calculated parameters of the S_0_–S_1_ CT transition for the complexes 1–3 are presented in the Table 3. As we expected, the B3LYP functional overestimates the energy of S_1_ state, while the CAM-B3LYP functional provides a better agreement with the measured diffusion reflection spectrum (Fig. 4). The intensity of the S_0_–S_1_ transition is expectedly very weak (oscillator strengths are of the 10^−4^ and 10^−3^ order calculated within B3LYP and CAM-B3LYP functionals, respectively) because of the strong space separation of the HOMO and LUMO.

**Table 3:** TD-DFT/B3LYP/6-31G(d) calculated energies, intensities and orbital assignments for the vertical S_0_–S_1_ electronic transitions of complexes 1–3 (the values obtained using CAM-B3LYP functional are presented in parentheses)

Complex	*E* (S_1_), nm	*f*	Assignment
1 (R = H)	357 (256)	0.0006 (0.0038)	HOMO–LUMO
2 (R = F)	356 (256)	0.0007 (0.0040)	HOMO–LUMO
3 (R = Me)	355 (256)	0.0006 (0.0036)	HOMO–LUMO

**Fig. 9:**
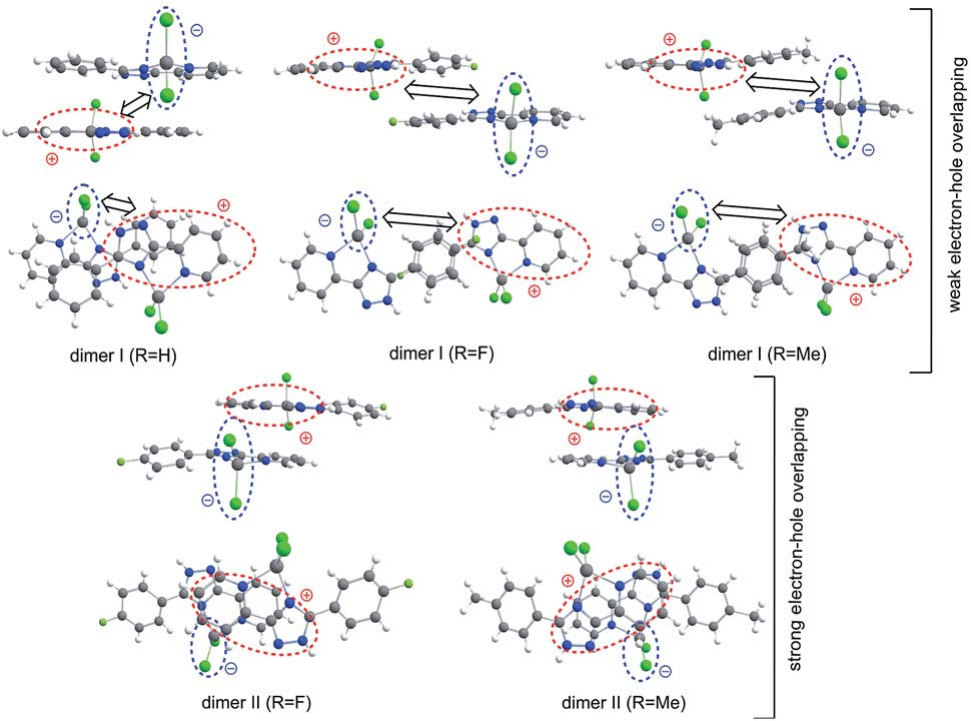
The structure of π-stacking dimers extracted from the X-ray crystallographic data. The type I dimer possesses a large space-separation between the hole and electron that implies a low probability of electron–hole emissive recombination, while type II dimers are characterized by a close localization of holes and electrons and a high recombination probability.

Moving to the type I dimers, one can see from Fig. 9 that the HOMO (e) and LUMO (h) are strongly space-separated, implying that the electron–hole (e–h) recombination is weakly probable. Moreover, the transition moment for such an emissive recombination should be close to zero. That is why the gradual decreasing of excitation energy does not lead to the emission in type I dimers and we can only observe the fast decay of molecular fluorescence moving far from resonance between the excitation energy and S_1_ energy of the single molecule complexes 1 and 5. Indeed, our TD-DFT/B3LYP/6-31G(d) calculations clearly show a very small red shift for the dimer absorption (363 nm for complex 1) relative to the single molecule spectrum (357 nm, Table 3) meaning an inefficient exchange coupling between the HOMOs and LUMOs of monomers in the type I dimers. Actually, type I dimers and corresponding monomers absorb and emit light at the close same wavelength and thus the electron–hole (e–h) recombination in dimer is hard to distinguish accounting also for the weak dipole probability of such process due to the strong space-separation of holes and electrons (Fig. 9 and S6†).

**Fig. 10:**
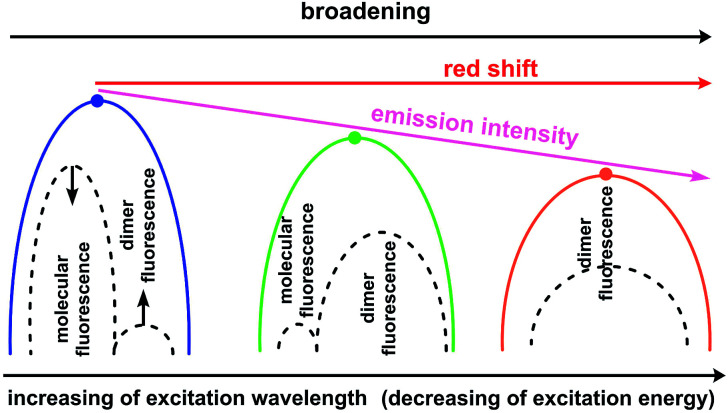
Schematic representation of intensity redistribution between the molecular and dimer fluorescence upon decrease of excitation energy for the solid state samples of compounds 2–4.

In contrast, type II dimers (complexes 2–4) are characterized by the close-lying e and h wave functions (Fig. 9) which correspond to a higher probability of e–h recombination and to a higher value of transition dipole moment for the emissive process. Upon the gradual decrease of excitation energy, the molecular fluorescence becomes weaker, while the red-shifted emission of molecular dimers II increases. Such an intensity redistribution is schematically presented in Fig. 10. The TD-DFT/B3LYP/6-31G(d) calculations for the separate dimers really show quite strong red shift of S_1_ energy level for the type II dimers of complexes 2 (380 nm) and 3 (384 nm) relative to the monomers (356 and 355 nm, respectively). At the same time S_1_ energy for type I dimers of complexes 2 and 3 also becomes considerably shifted (377 and 392 nm, respectively). It means the efficient exchange coupling between the HOMO and LUMO wavefunctions of monomers within type I/II dimers of F- and Me-substituted complexes 2 and 3. Such strong red shift also means that it is clearly possible to populate separately S_1_ state of monomers by higher excitation energies and type I/II dimer S_1_ states by smaller excitation pulses. The emission response from monomers and dimers thus is also clearly different and we can observe the excitation-dependent fluorescence (Table 2, Fig. 6). But the probability of the electron–hole (e–h) recombination depends mostly on the space separation of the corresponding HOMO and LUMO wave-functions, that are schematically shown in Fig. 9. The real HOMO and LUMO patterns are presented in Fig. S6.† One should comment that only the crossing coupling of HOMO and LUMO fragments should be considered in the case of orbitals presented in Fig. S6† to be consistent with the Fig. 9. It is because of the HOMO and LUMO wave-functions of the individual dimers actually represent the doubly multiplied HOMO and LUMO wave-functions of single molecules, while in the real crystal we deal with the periodically ordered structure and electron–hole recombination occurs between the molecular-localized charge carriers.

The theoretical calculations are in good agreement with the experimental excitation spectra measured for complex 3 monitored at 420 and 563 nm emission wavelengths respectively (Fig. S7†). In the first case excitation spectra contain broad band with maximum at 348 nm (similar spectra was found for 1). From the other hand monitoring the long-wavelength emission band we observe multicomponent band with main maximum at 409 nm assigned to type II dimers activation.

One can see from Fig. 10 that the experimentally observed gradual red-shift of emission maximum upon decreasing of excitation energy originates from the additive effect between two emission bands (molecular and dimer emission). A higher population of the excited state dimers II provides an enchantment of the long-wavelength band that is expectedly accompanied by a decrease of the S_1_ state population of separate molecules because of mismatch between S_1_ energy and excitation energy. For sure, the emission intensity of the e–h recombination within dimers II is weaker comparing with the molecular fluorescence that correspond to the decrease of overall emission intensity and quantum yield. However, due to the similar degree of space separation for the single molecule and dimers of type II the ratio between the molecular fluorescence and dimer II fluorescence decreases slowly and therefore we can observe high quantum yield values upon the small excitation energies, comparable with those upon the high excitation energies (Table 2).

## Conclusions

In summary, five Zn(ii) complexes with different pyridyltriazole ligands were synthesized and the effect of substitution on the structural as well as spectroscopic properties were examined. It was found that slight deviations in molecular structure induce considerable changes in the supramolecular architecture. The emission maximum wavelengths of complexes 1–5 can be tuned in the range of 396–436 nm through the *para*-substitution in the phenyl group. The supramolecular differences lead to the realisation of different emission properties at different excitation energies. The proposed model for this phenomenon comprises a double-channel fluorescence originating from the single molecules and strongly coupled dimers in excellent agreement with the luminescence decay kinetics measurements. These unique properties make the ground for complexes 1–5 to become versatile luminochromic material despite their simple molecular structure.

## Conflicts of interest

There are no conflicts to declare.

## Supplementary Material

RA-009-C9RA02491C-s001

RA-009-C9RA02491C-s002
