# Supplementary material for: Structure and excitation-dependent emission of novel zinc complexes with pyridyltriazoles
Source: RSC Adv. 2019 Jul 16;9(38):22143–52. doi: 10.1039/c9ra02491c (PMC9066697; doi:10.1039/c9ra02491c)
Supplement: RA-009-C9RA02491C-s001 [file RA-009-C9RA02491C-s001.pdf]

Electronic Supplementary Material (ESI) for RSC Advances.
This journal is © The Royal Society of Chemistry 2019

Supplementary Information

Structure and excitation-dependent emission of novel Zinc complexes with pyridyltriazoles

Alexey Gusev,*[a] Elena Braga[a], Ekaterina Zamnius[a], Mikhail Kiskin[a], Mariya Kryukova[b], Alina Baryshnikova,[c] Boris Minaev,[c] Gleb Baryshnikov,[c,d] Hans Ågren[d,e], Wolfgang Linert*[f]

[a]*N.S. Kurnakov Institute of General and Inorganic Chemistry, Russian Academy of Sciences, Moscow, 119991, Russia.*
[b]*Institute of Chemistry, Saint PetersburgStateUniversity, Universitetskaya Nab. 7/9, Saint Petersburg, 199034, Russia.*
[c] *Department of Chemistry and Nanomaterials Science, BohdanKhmelnytsky National University, 18031, Cherkasy, Ukraine*
[d] *Division of Theoretical Chemistry and Biology, School of Engineering Sciences in Chemistry, Biotechnology and Health, KTH Royal Institute of Technology, 10691, Stockholm, Sweden*
[e] *Tomsk State University, 36 Lenin Avenue, Tomsk, Russia*
[f] *Institute for Applied Synthetic Chemistry, Vienna University of Technology, Getreidemarkt 9/163, A-1060 Vienna, Austria*

Experimental Procedure

Synthesis of 5-(4-R-phenyl)-3-(pyridin-2-yl)-1,2,4-triazoles L1-L5 (general method)

Sodium metal (0.8 g) was added carefully to 35 cm^3 of methanol followed by addition of 2-pyridine carbonitrile (5.5 g, 52 mmol). The solution was left undisturbed for 30 min. Related carboxylic acid hydrazides (benzoic, p-toluic, p-anisic, p-fluorobenzoic, isonicotinic) (40 mmol) were added to the solution followed by addition of 1 ml of acetic acid. The reaction mixture was stirred and refluxed for 2 hours. A light yellow solid was formed during the reaction, filtered off upon cooling under vacuum and air dried for one day. The semiproducts were dissolved in 40 cm^3 of ethyleneglycol and refluxed for 3 h. On cooling overnight the white target ligand precipitated and was collected by vacuum filtration, followed by washing with 20 cm^3 of cold water. The resulting triazoles were recrystallized from MeOH-water.

5-(4-phenyl)-3-(pyridin-2-yl)-1,2,4-triazole Yield: 8.1 g (91 %). Anal. Calcd (%) for $C_{13}H_{10}N_4$: C, 70.25; H, 4.53; N, 25.21. Found: C, 70.19; H, 4.38; N, 25.24. ^1H NMR (400 MHz, DMSO-d^6): δ 14.15 (s, 1H), 8.68 (d, 1H), 8.11 (m, 3H), 8.02 (t, 1H), 7.48 (t, 1H), 7.32 (m, 3H). FT-IR bands (cm^{-1}): 3069 (w), 1601 (s), 1566 (s), 1484 (s), 1458 (s), 1412 (s), 1150 (s), 1010 (s), 726(s).

5-(4-F-phenyl)-3-(pyridin-2-yl)-1,2,4-triazole Yield: 8.6 g (90 %). Anal. Calcd (%) for $C_{13}H_9FN_4$: C, 64,99; H, 3.77; N, 23.32. Found: C, 65.02; H, 3.59; N, 23.24. ^1H NMR (400 MHz, DMSO-d6): δ 14.12 (s, 1H), 8.70 (d, 1H), 8.19 (d, 1H), 8.08 (d, 2H), 8.01 (dd, 1H), 7.51 (dd, 1H), 7.45 (d, 2H). FT-IR bands (cm^{-1}): 3070 (w), 1600 (s), 1558 (s), 1528 (m), 1483 (s), 1414 (s), 1211 (s), 1153 (s), 1011 (s), 753(s).

5-(4-methylphenyl)-3-(pyridin-2-yl)-1,2,4-triazole Yield: 7.9 g (84 %). Anal. Calcd (%) for $C_{14}H_{12}N_4$: C, 71.17; H, 5.21; N, 23.71. Found: C, 71.21; H, 5.06; N, 23.77. ^1H NMR (400 MHz, DMSO-d6): δ 14.18 (s, 1H), 8.65 (d, 1H), 8.15 (d, 1H), 8.01 (dd, 1H), 7.98 (d, 2H), 7.50 (dd, 1H), 7.34 (d, 2H), 2.39 (s, 3H). FT-IR bands (cm^{-1}): 3029 (w), 1600 (s), 1572 (m), 1555 (m), 1479 (s), 1411 (m), 1150 (m), 1008 (s), 745(s).

5-(4-methoxyphenyl)-3-(pyridin-2-yl)-1,2,4-triazole Yield: 9.5 g (94 %). Anal. Calcd (%) for $C_{14}H_{12}N_4O$: C, 66.65; H, 4.79; N, 22.21. Found: C, 66.52; H, 4.55; N, 22.17. ^1H NMR (400 MHz, DMSO-d6): δ 14.04 (s, 1H), 8.71 (d, 1H), 8.17 (d, 1H), 8.04 (m, 3H), 7.56 (t, 1H), 7.08 (d, 2H), 3.82 (s, 3H). FT-IR bands (cm^{-1}): 3069 (w), 1602 (s), 1573 (m), 1535 (m), 1473 (s), 1417 (m), 1248 (m), 1151 (s), 1009 (s), 753(s).

5-(pyridine-4-yl)-3-(pyridin-2-yl)-1,2,4-triazole Yield: 7.5 g (85 %). Anal. Calcd (%) for $C_{12}H_9N_5$: C, 64.56; H, 4.06; N, 31.37. Found: C, 64.49; H, 3.98; N, 31.36. ^1H NMR (400 MHz, DMSO-d6): δ 14.28 (s, 1H), 8.71 (d, 2H), 8.64 (d, 1H), 8.12 (d, 1H), 8.01 (m, 3H), 7.53 (dd, 1H). FT-IR bands (cm^{-1}): 3069 (w), 1600 (s), 1564 (s), 1482(s), 1458(s), 1411(s), 1150 (s), 1010 (s), 724(s).

Table S1. Crystal data and structure refinements for **1-3** and **5**.

Parameter	1	2	3	5
CCDC number	1905545	1905547	1905548	1905546
Formula	$C_{26}H_{20}Cl_4N_8Zn_2$	$C_{13}H_9Cl_2FN_4Zn$	$C_{14}H_{12}Cl_2N_4Zn$	$C_{12}H_9Cl_2N_5Zn$
Crystal system	Monoclinic	Triclinic	Triclinic	Monoclinic
Space group	$P2_1/m$	$P\bar{1}$	$P\bar{1}$	$P2_1/m$
a, Å<br>b, Å<br>c, Å	8.859(3)<br>7.160(2)<br>11.567(4)	7.2313(2)<br>14.1852(4)<br>15.3646(4)	8.0697(14)<br>8.3754(15)<br>12.679(3)	8.8612(4)<br>7.1511(3)<br>11.5516(5)
α °<br>β°<br>γ°	90<br>95.287(11)<br>90	74.608(2)<br>79.912(2)<br>76.548(2)	84.525(7)<br>78.822(6)<br>63.230(5)	90<br>95.301(4)<br>90
V, Å ^{3}<br>Z	730.5(4)<br>1	1466.99(7)<br>4	750.6(2)<br>2	728.86(5)<br>2
λ, Å	0.71073	1.54184	0.71073	1.54184
μ mm^{-1}	2.040	5.763	1.989	5.663
Parameters	118	397	195	150
No. refl. unique	1595	10017	3036	4990
No. refl. I > 2σ(I)	1304	9641	2931	4354
GOF	0.992	1.119	1.166	1.105
R(I > 2σ(I))	0.0549	0.0835	0.0223	0.0428
wR_2	0.1477	0.2391	0.0709	0.1492

Table S2. Topological characteristics of the non-covalent interactions responsible for the binding of complex 1 (R=H) with all neighbouring molecules within packing fragment (complex 1)$_9$.

Bonds	$\rho(\mathbf{r})$. $e\cdot a_o^{-3}$ *	$v(\mathbf{r})$. a.u.	$g(\mathbf{r})$. a.u.	$h_e(\mathbf{r})$. a.u.	$\nabla^2\rho(\mathbf{r})$. $e\cdot a_o^{-5}$	ε	E (EML) kcal mol^{-1}
H54-Cl149	0.0041	-0.0017	0.0027	0.0010	0.0148	0.1174	-0.55
H54-Cl120	0.0041	-0.0017	0.0027	0.0010	0.0148	0.1174	-0.55
H56-N4	0.0119	-0.0077	0.0087	0.0010	0.0388	0.0548	-2.40
C51-C165	0.0039	-0.0017	0.0023	0.0006	0.0115	5.5359	-0.55
C51-C225	0.0039	-0.0017	0.0023	0.0006	0.0115	5.5359	-0.55
C57-N154	0.0037	-0.0019	0.0024	0.0005	0.0114	0.8403	-0.60
C57-N214	0.0037	-0.0019	0.0024	0.0005	0.0114	0.8403	-0.60
C48-C226	0.0036	-0.0017	0.0023	0.0006	0.0114	0.8247	-0.54
C48-C166	0.0036	-0.0017	0.0023	0.0006	0.0114	0.8247	-0.54
H58-H14	0.0011	-0.0004	0.0007	0.0003	0.0039	1.0270	-0.14
H50-Cl239	0.0060	-0.0028	0.0040	0.0012	0.0206	0.1032	-0.87
H50-Cl180	0.0060	-0.0028	0.0040	0.0012	0.0206	0.1032	-0.87
C47-C227	0.0031	-0.0015	0.0021	0.0006	0.0109	3.1915	-0.46
C47-C167	0.0031	-0.0015	0.0021	0.0006	0.0109	3.1915	-0.46
H36-Cl180	0.0105	-0.0068	0.0085	0.0017	0.0409	0.1912	-2.12
H36-Cl239	0.0105	-0.0068	0.0085	0.0017	0.0409	0.1912	-2.12
N34-C237	0.0036	-0.0019	0.0023	0.0005	0.0113	1.0471	-0.59
N34-C177	0.0036	-0.0019	0.0023	0.0005	0.0113	1.0471	-0.59
C46-C228	0.0036	-0.0017	0.0023	0.0006	0.0116	0.6707	-0.55
C46-C168	0.0036	-0.0017	0.0023	0.0006	0.0116	0.6707	-0.55
N34-H86	0.0119	-0.0076	0.0086	0.0010	0.0385	0.0547	-2.39
Cl59-H170	0.0062	-0.0028	0.0040	0.0012	0.0207	0.0549	-0.88
Cl59-H188	0.0053	-0.0024	0.0037	0.0013	0.0200	0.4185	-0.74

Cl59-H156	0.0106	-0.0068	0.0086	0.0017	0.0413	0.1399	-2.14
Cl59-H114	0.0044	-0.0018	0.0028	0.0010	0.0151	0.0766	-0.58
Cl59-H12	0.0044	-0.0019	0.0029	0.0010	0.0156	0.3043	-0.58
Cl60-H248	0.0053	-0.0024	0.0037	0.0013	0.0200	0.4185	-0.74
Cl60-H230	0.0062	-0.0028	0.0040	0.0012	0.0207	0.0549	-0.88
Cl60-H216	0.0106	-0.0068	0.0086	0.0017	0.0413	0.1399	-2.14
Cl60-H144	0.0044	-0.0018	0.0028	0.0010	0.0151	0.0766	-0.58
Cl60-H12	0.0044	-0.0019	0.0029	0.0010	0.0156	0.3043	-0.58
C45-C231	0.0040	-0.0018	0.0023	0.0006	0.0115	5.2333	-0.56
C45-C171	0.0040	-0.0018	0.0023	0.0006	0.0115	5.2333	-0.56
H44-H88	0.0011	-0.0004	0.0007	0.0003	0.0039	1.4850	-0.13
H42-Cl89	0.0044	-0.0019	0.0029	0.0010	0.0156	0.2994	-0.58
H42-Cl90	0.0044	-0.0019	0.0029	0.0010	0.0156	0.2994	-0.58
H38-Cl269	0.0052	-0.0023	0.0036	0.0013	0.0198	0.4152	-0.73
H38-Cl210	0.0052	-0.0023	0.0036	0.0013	0.0198	0.4152	-0.73
Sum	0.1993	-0.1030	0.1381	0.0352	-	-	-32.31

Table S3. Topological characteristics of the non-covalent interactions responsible for the binding of complex 2 (R=F) with all neighbouring molecules within packing fragment (complex 2)$_{11}$.

Bonds	$\rho(\mathbf{r})$. $e \cdot a_o^{-3}$ *	$v(\mathbf{r})$. a.u.	$g(\mathbf{r})$. a.u.	$h_e(\mathbf{r})$. a.u.	$\nabla^2 \rho(\mathbf{r})$. $e \cdot a_o^{-5}$	ε	E (EML) kcal mol^{-1}
H141-Cl153	0.0087	-0.0048	0.0065	0.0017	0.03	0.05	-1.50
H132-Cl2	0.0058	-0.0026	0.0039	0.0013	0.02	0.07	-0.82
C131-C10	0.0048	-0.0020	0.0028	0.0007	0.01	0.32	-0.64
C131-N65	0.0049	-0.0025	0.0030	0.0005	0.01	1.07	-0.78
C138-N67	0.0049	-0.0023	0.0029	0.0006	0.01	0.74	-0.73
H139-H170	0.0007	-0.0003	0.0004	0.0002	0.00	3.63	-0.08
H139-N278	0.0038	-0.0018	0.0025	0.0007	0.01	0.04	-0.57
H134-Cl63	0.0062	-0.0028	0.0043	0.0015	0.02	0.62	-0.88
C130-C70	0.0045	-0.0022	0.0029	0.0007	0.01	11.78	-0.69
C130-C11	0.0049	-0.0021	0.0028	0.0007	0.01	0.33	-0.66
N127-C78	0.0048	-0.0023	0.0029	0.0006	0.01	0.76	-0.72
N127-H260	0.0048	-0.0025	0.0033	0.0009	0.02	0.03	-0.77
N125-C71	0.0050	-0.0026	0.0031	0.0005	0.01	0.94	-0.81
N128-Cl303	0.0051	-0.0027	0.0038	0.0011	0.02	0.78	-0.84
H129-Cl242	0.0182	-0.0142	0.0155	0.0012	0.07	0.05	-4.47
C137-F34	0.0031	-0.0018	0.0029	0.0010	0.02	0.15	-0.58
Cl122-H300	0.0139	-0.0099	0.0115	0.0016	0.05	0.07	-3.10
Cl122-H12	0.0057	-0.0026	0.0039	0.0013	0.02	0.09	-0.83
Cl122-H56	0.0034	-0.0013	0.0021	0.0007	0.01	0.07	-0.42
Cl122-H286	0.0057	-0.0025	0.0038	0.0013	0.02	0.15	-0.80
Cl123-H286	0.0029	-0.0011	0.0017	0.0006	0.01	0.21	-0.36
Cl123-N276	0.0059	-0.0031	0.0038	0.0006	0.02	0.07	-0.99
Cl123-H74	0.0062	-0.0029	0.0044	0.0015	0.02	0.61	-0.90
Cl123-H239	0.0051	-0.0023	0.0034	0.0011	0.02	0.02	-0.71
Cl123-F184	0.0049	-0.0032	0.0045	0.0013	0.02	0.14	-1.02
Cl123-H198	0.0032	-0.0012	0.0020	0.0007	0.01	0.69	-0.39
Cl123-H116	0.0004	-0.0001	0.0002	0.0001	0.00	0.13	-0.04
C136-C59	0.0057	-0.0026	0.0033	0.0007	0.02	1.38	-0.81
H148-Cl303	0.0094	-0.0054	0.0072	0.0018	0.04	0.03	-1.70
H148-Cl242	0.0040	-0.0017	0.0026	0.0009	0.01	0.31	-0.53

H146-Cl32	0.0034	-0.0013	0.0020	0.0007	0.01	0.09	-0.41
F124-Cl183	0.0040	-0.0025	0.0036	0.0011	0.02	0.01	-0.78
F124-C47	0.0031	-0.0018	0.0028	0.0010	0.02	0.15	-0.56
C142-C117	0.0060	-0.0025	0.0033	0.0008	0.02	0.61	-0.79
C149-C46	0.0058	-0.0026	0.0034	0.0007	0.02	1.25	-0.83
H150-Cl183	0.0020	-0.0008	0.0012	0.0004	0.01	1.95	-0.24
H150-Cl82	0.0056	-0.0026	0.0037	0.0012	0.02	0.03	-0.81
Sum	0.1964	-0.1037	0.1378	0.0341			-32.54

Table S4. Topological characteristics of the non-covalent interactions responsible for the binding of complex 3 (R=Me) with all neighbouring molecules within packing fragment (complex 3)$_{10}$.

Bonds	$\rho(\mathbf{r})$. $e\cdot a_o^{-3}$ *	$v(\mathbf{r})$. a.u.	$g(\mathbf{r})$. a.u.	$h_e(\mathbf{r})$. a.u.	$\nabla^2\rho(\mathbf{r})$. $e\cdot a_o^{-5}$	ε	E (EML) kcal mol^{-1}
Cl101-C252	0.0057	-0.0027	0.0039	0.0012	0.0204	0.61	-0.86
H255-H114	0.0033	-0.0015	0.0024	0.0009	0.0130	0.11	-0.46
H253-N169	0.0029	-0.0016	0.0022	0.0006	0.0111	5.43	-0.50
H253-Cl168	0.0023	-0.0008	0.0013	0.0004	0.0069	0.97	-0.26
H254-N171	0.0046	-0.0027	0.0035	0.0008	0.0173	0.73	-0.85
H246-H114	0.0038	-0.0016	0.0025	0.0010	0.0140	3.64	-0.50
H246-H123	0.0033	-0.0014	0.0023	0.0009	0.0129	0.18	-0.45
H246-Cl36	0.0048	-0.0021	0.0032	0.0011	0.0171	0.08	-0.65
C247-C176	0.0044	-0.0021	0.0027	0.0006	0.0134	6.88	-0.65
H249-Cl168	0.0061	-0.0028	0.0040	0.0012	0.0208	0.06	-0.86
C243-C184	0.0053	-0.0023	0.0029	0.0006	0.0142	2.30	-0.73
C250-C177	0.0052	-0.0023	0.0029	0.0006	0.0141	2.39	-0.71
C242-C181	0.0045	-0.0021	0.0027	0.0007	0.0136	4.62	-0.65
H251-Cl299	0.0052	-0.0023	0.0033	0.0011	0.0176	0.07	-0.71
C241-C160	0.0045	-0.0021	0.0028	0.0007	0.0140	0.15	-0.64
N237-Cl299	0.0043	-0.0021	0.0029	0.0008	0.0147	1.19	-0.67
N237-H188	0.0046	-0.0027	0.0035	0.0008	0.0174	0.77	-0.85
H238-Cl300	0.0158	-0.0117	0.0129	0.0012	0.0566	0.03	-3.68
C240-H187	0.0029	-0.0016	0.0022	0.0006	0.0112	45.98	-0.50
N236-H231	0.0025	-0.0012	0.0018	0.0006	0.0097	3.40	-0.38
N236-H229	0.0027	-0.0013	0.0020	0.0007	0.0107	0.94	-0.41
Cl233-C120	0.0057	-0.0028	0.0039	0.0012	0.0205	0.54	-0.86
Cl223-H29	0.0061	-0.0029	0.0045	0.0016	0.0244	0.45	-0.91
Cl233-H165	0.0085	-0.0043	0.0064	0.0020	0.0337	0.54	-1.36
Cl233-H27	0.0085	-0.0046	0.0065	0.0019	0.0334	0.15	-1.44
Cl234-H97	0.0067	-0.0031	0.0045	0.0013	0.0233	0.09	-0.99
Cl234-H187	0.0023	-0.0009	0.0013	0.0004	0.0070	0.87	-0.27
Cl234-H48	0.0048	-0.0021	0.0031	0.0011	0.0168	0.06	-0.64
Cl234-H183	0.0061	-0.0028	0.0040	0.0012	0.0209	0.06	-0.87
N239-C141	0.0030	-0.0016	0.0021	0.0005	0.0103	1.49	-0.50
N236-H324	0.0013	-0.0005	0.0009	0.0004	0.0051	0.68	-0.16
C257-C94	0.0051	-0.0022	0.0029	0.0007	0.0146	0.85	-0.70
H258-Cl2	0.0086	-0.0047	0.0065	0.0019	0.0336	0.14	-1.47
H260-Cl2	0.0060	-0.0029	0.0045	0.0016	0.0245	0.46	-0.92
C259-C142	0.0045	-0.0021	0.0028	0.0007	0.0140	0.14	-0.65
C259-C92	0.0051	-0.0022	0.0029	0.0007	0.0144	0.84	-0.69
H262-Cl69	0.0065	-0.0031	0.0045	0.0014	0.0235	0.09	-0.97
H262-H287	0.0012	-0.0004	0.0008	0.0003	0.0043	0.23	-0.13
H262-N203	0.0027	-0.0013	0.0020	0.0007	0.0107	0.96	-0.41

H264-Cl134	0.0086	-0.0043	0.0064	0.0020	0.0335	0.52	-1.36
H264-N203	0.0025	-0.0012	0.0018	0.0006	0.0097	4.72	-0.37
H264-H231	0.0042	-0.0018	0.0031	0.0013	0.0177	0.16	-0.58
H264-Cl134	0.0086	-0.0043	0.0064	0.0020	0.0335	0.52	-1.36
Sum	0.2154	-0.1070	0.1498	0.0428			-33.58

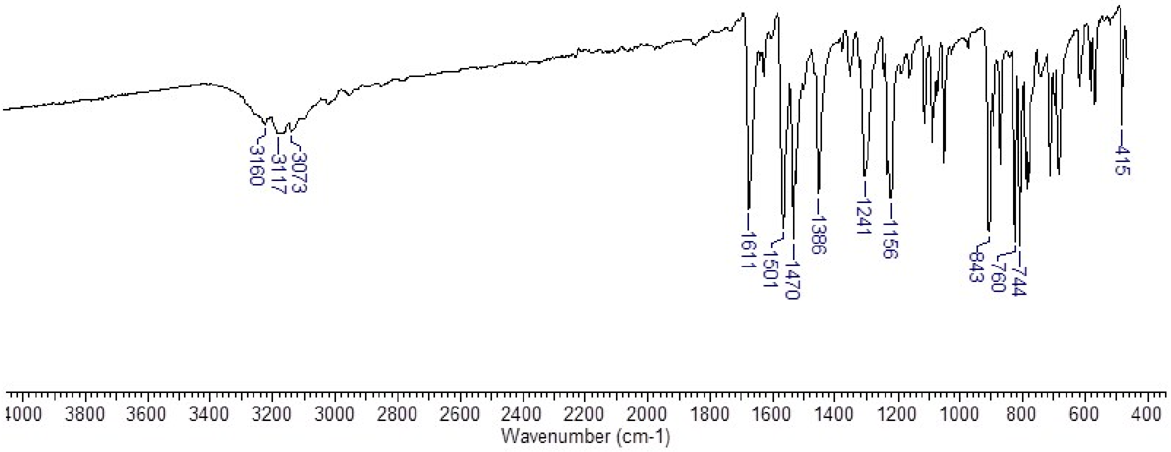

Figure S1 – IR-spectra of complex 2.

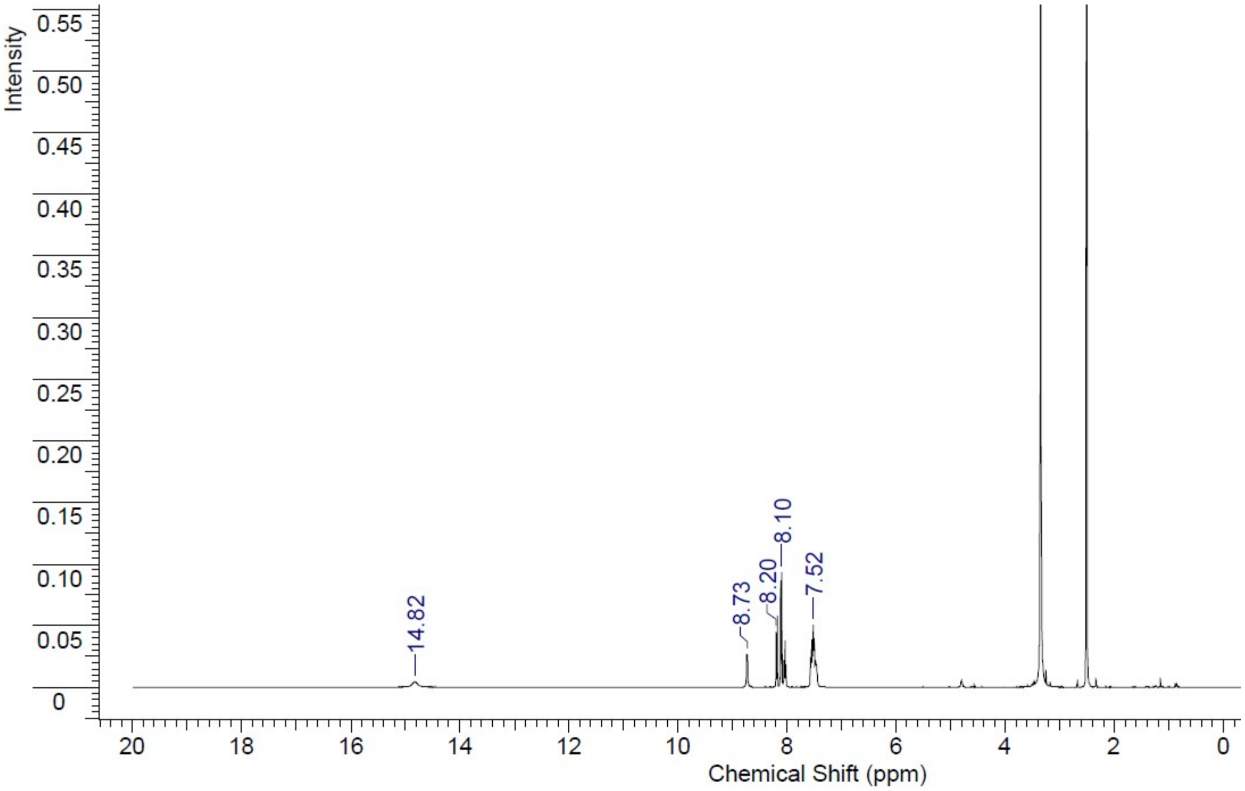

Figure S2 – ^{1}H-NMR-spectra of complex 2.

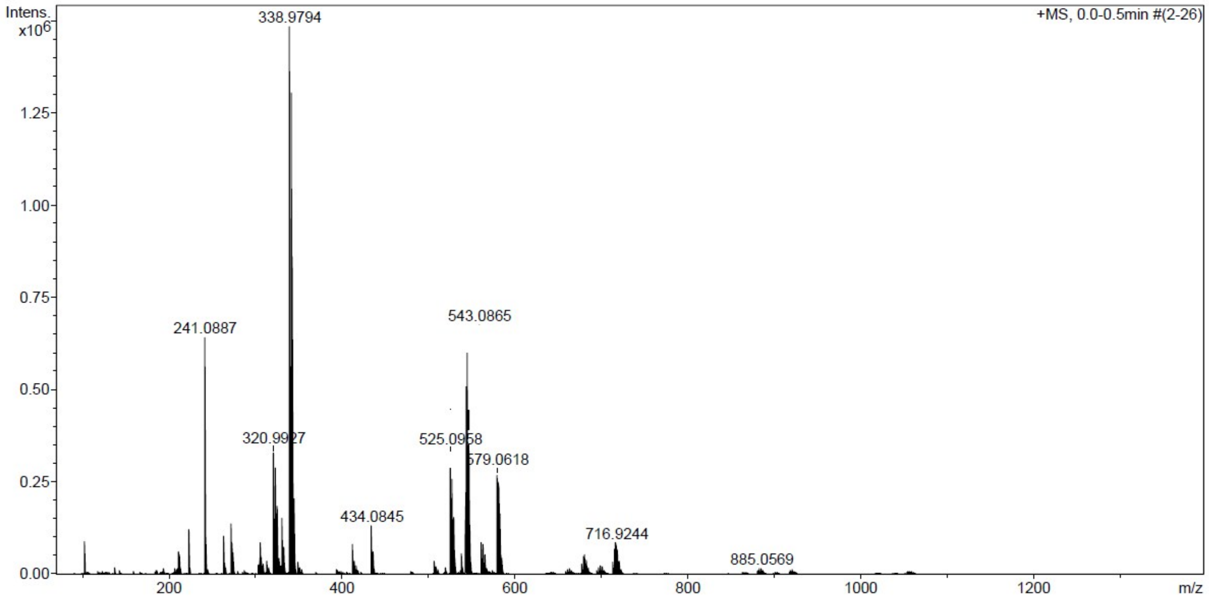

Figure S3 – ES-mass-spectra of complex 2.

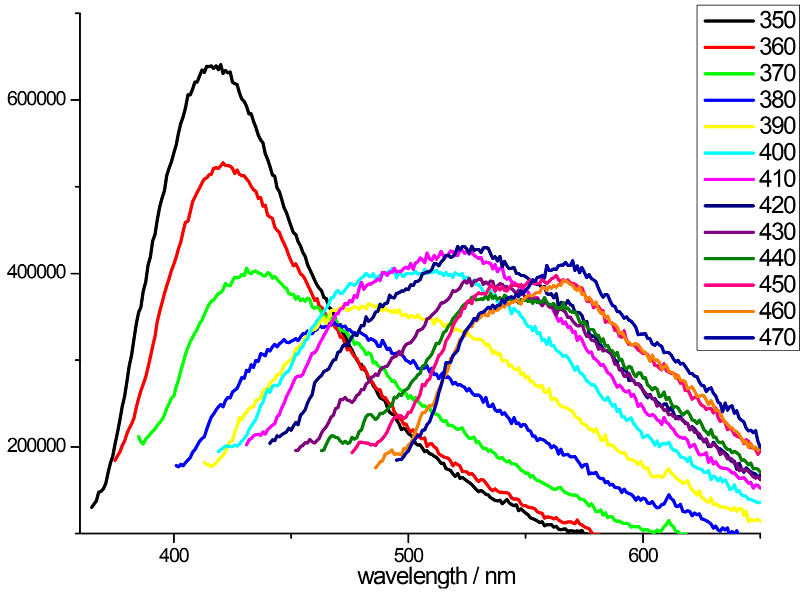

Figure S4. Emission spectra of 3 in the solid state at different excitation.

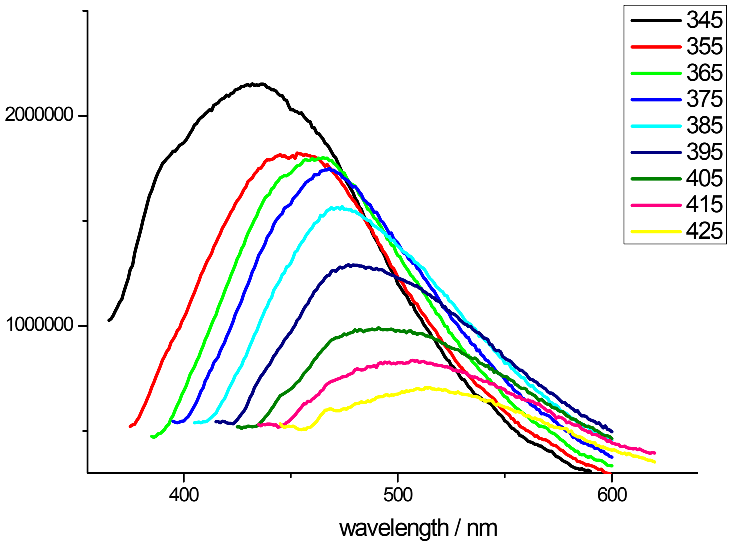

Figure S5. Emission spectra of 4 in the solid state at different excitation.

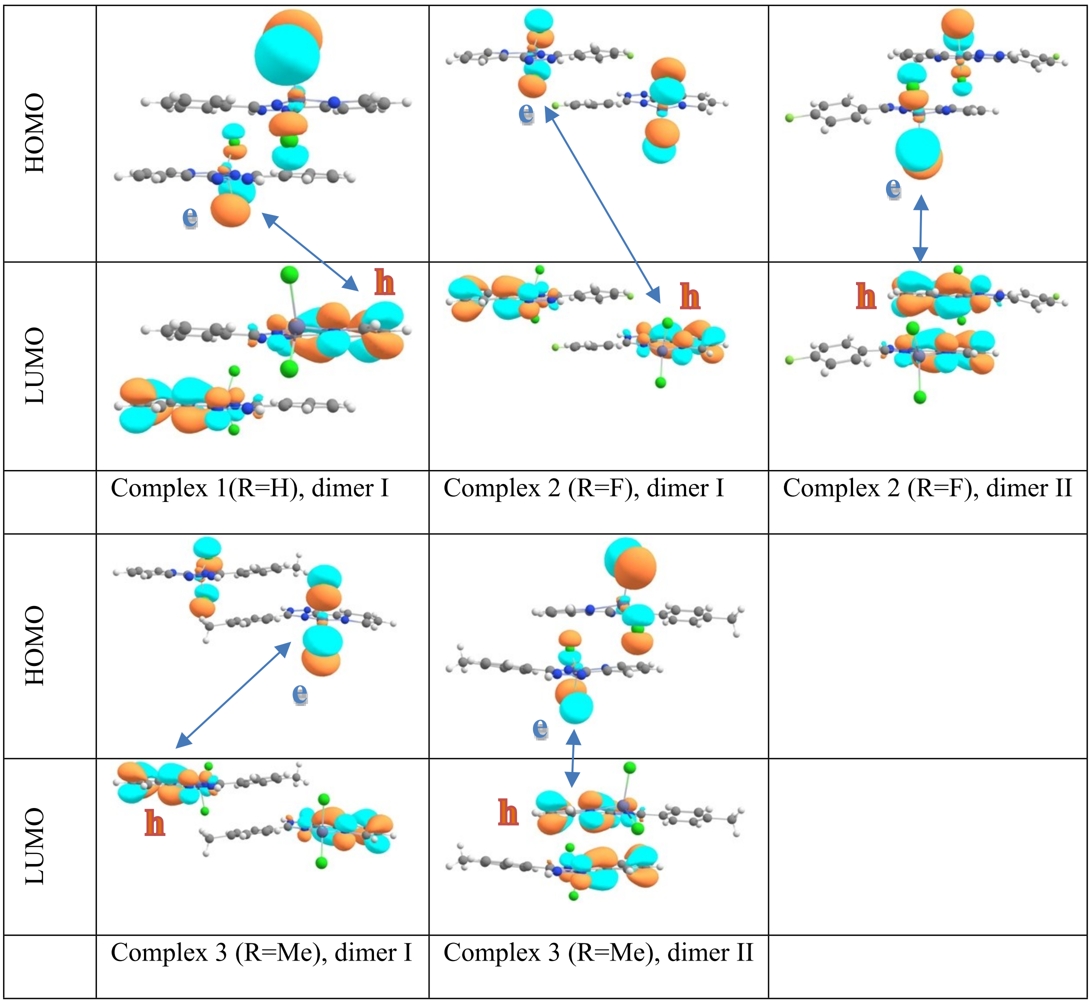

Figure S6. The shape of the frontier molecular orbitals for the individual (isolated) dimers of type I and II for the complexes 1-3 calculated by the B3LYP/6-31G(d) method (the control value of isosurface is 0.03 a.u.).

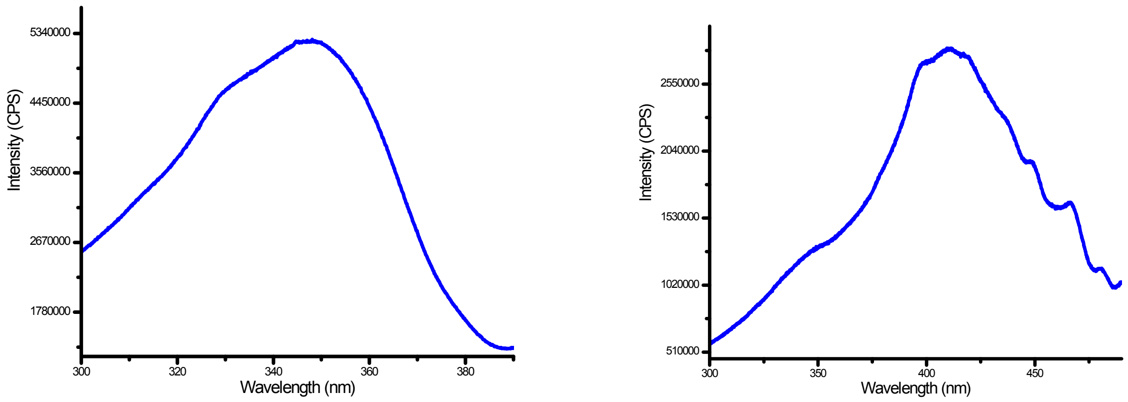

Figure S7. excitation spectra monitored at 420 (left) and 563 nm (right) emission wavelengths for complex 3.
